# Defect-Tailored Ag_2_SeO_3_: Morphology and Function Tuned by pH-Driven Sonochemical Synthesis

**DOI:** 10.1021/acsomega.6c00644

**Published:** 2026-03-19

**Authors:** Henrique Moreno, Giovanna A. Grasser, Marcio D. Teodoro, Marcelo Assis, Elson Longo

**Affiliations:** † CDMF-LIEC, 67828 Federal University of São Carlos (UFSCar), São Carlos 13565-905, Brazil; ‡ Department of Physics, Federal University of São Carlos (UFSCar), São Carlos 13565-905, Brazil; § Department of Biosciences, 28105 Federal University of São Paulo (UNIFESP), Santos, São Paulo 11015-020, Brazil

## Abstract

Ag_2_SeO_3_ particles were synthesized via a pH-driven sonochemical route (pH = 2, 5, and 12) to elucidate how defect chemistry and morphology govern photocatalytic and antimicrobial performance. X-ray diffraction and Raman spectroscopy confirmed the formation of monoclinic Ag_2_SeO_3_, while increasing synthesis pH progressively destabilized the lattice, inducing preferred orientation, short-range disorder, and partial segregation of metallic Ag under alkaline conditions. XPS revealed a gradual shift in silver speciation from Ag^+^-dominated lattices toward mixed Ag^+^/Ag^0^ states and reduced lattice oxygen at high pH, consistent with enhanced defect density. Morphological evolution from microrods (pH 2) to shortened rods (pH 5) and sheet-like particles (pH 12) was observed, driven by pH-dependent nucleation and growth kinetics. Optical analyses showed comparable band gaps (∼3.5–3.7 eV), while photoluminescence deconvolution evidenced defect-mediated suppression of charge recombination, particularly for alkaline-synthesized Ag_2_SeO_3_. Consequently, the pH 12 sample exhibited superior photocatalytic activity toward ciprofloxacin degradation (∼98% under UV irradiation), governed predominantly by ^•^O_2_H and ^1^O_2_ species, as demonstrated by scavenger and probing experiments. Toxicity assays confirmed that photocatalytic residues were nontoxic toward *Staphylococcus aureus* and *Lactuca sativa*, despite limited mineralization. Antibacterial tests revealed tunable, pH-dependent selectivity: acidic samples favored Gram-positive inhibition, while alkaline samples enhanced Gram-negative activity via synergistic ROS generation and Ag^+^ release. Cytotoxicity and intracellular redox analyses demonstrated concentration-dependent oxidative stress, underscoring the importance of post-treatment separation. Overall, this study establishes pH modulation during sonochemical synthesis as an effective strategy to engineer defect landscapes and multifunctionality in Ag_2_SeO_3_ for environmental remediation and antimicrobial applications.

## Graphical abstract

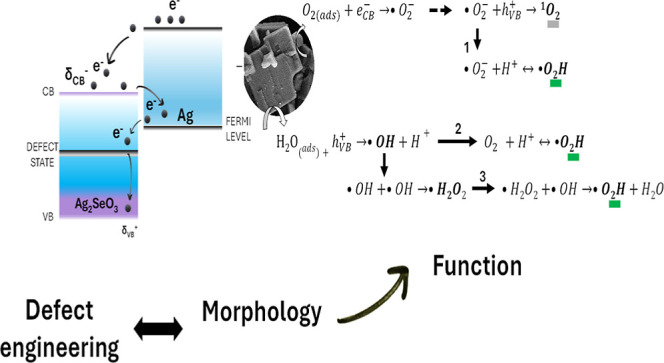

## 1 Introduction

In the last decades, although essential to life, water resources have been severely limited due to pollution resulting from growing urbanization, industrialization, and agricultural production. The most contamination is associated with pesticides, pharmaceuticals, surfactants, and microplastics, among others, which reach aquatic ecosystems mainly through domestic, hospital, agricultural, and industrial wastewater.^1–3^ These so-called emerging contaminants (ECs) are not yet regulated for their presence in effluents. Among ECs, persistent organic pollutants (POPs) deserve special attention, as they have been found even in Arctic ice caps. Antibiotics are a class of POPs that deserve particular attention.

Ciprofloxacin (CIP, C_17_H_18_FN_3_O_3_), a second-generation quinolone antibiotic, is widely administered to both humans and animals.^4^ Quinolone antibiotics account for around 90% of antibiotic consumption in Europe, of which CIP accounts for ∼50%.^5^ A systematic review by Kelly and Brooks^6^ reported its worldwide spread in Africa, Asia, Europe, North America, and South America. CIP has broad-spectrum antibacterial activity, being effective against Gram-positive and Gram-negative bacteria and several infections. However, CIP cannot be efficiently metabolized in the human body—about 75% of it is excreted—ending up in the environment.^7,8^ CIP persists in the aquatic environment, threatening the ecosystem and contributing to the rise of bacterial resistance.^9,10^ Rising antimicrobial resistance poses a significant threat to global health, driving demand for advanced antimicrobial solutions.

Given the degradation of CIP in effluents, traditional wastewater treatment has proven ineffective for removing antibiotics through ordinary physical, chemical, and biological treatment.^11^ To that end, photocatalytic processes are an efficient, cost-effective, and sustainable approach.^2^ In recent years, researchers have worked extensively to improve the efficiency of metal oxides toward the photodegradation of POPs. Silver-based compounds^12–17^ have drawn the attention of the scientific community due to enhanced visible light absorption, excellent quantum efficiency, and charge separation efficiency, etc.^18^

Selenium-based compounds have emerged as effective bactericidal agents, especially when combined with silver, due to their well-established antimicrobial activity.^19^ Recent studies highlight that Ag/Se-based materials exhibit robust, broad-spectrum antimicrobial activity at remarkably low concentrations, paired with excellent biocompatibility and stability, owing to their carefully engineered nanoscale structures.^20^ Mirzaei et al.^19^ highlighted the applications of selenium-based nanomaterials, ranging from antibiofilm to antiviral functions, associated with robust physicochemical stability and tunable ROS-mediated mechanisms. Microbiologically synthesized Ag_2_Se nanoparticles (∼12 nm) effectively inhibited key clinical pathogens (i.e., *Escherichia coli*, *Pseudomonas aeruginosa*, *Staphylococcus aureus*, *Bacillus. subtilis*, and *Candida. albicans*) in biofilms without cytotoxic effects on mammalian cells.^20^ Moreover, comparative studies indicate that selective reactive oxygen species (ROS) generated by Ag–Se based-materials outer membranes affect redox homeostasis, enhancing their antimicrobial activity across bacteria, fungi, and viruses.^21^ The study by Ghoniem et al.^22^ reported superior bactericidal efficacy, producing inhibition zones up to 32 mm (outperforming standard antibiotics such as cefotaxime) and MICs of around 160 μg/mL for *E. coli* and *S. aureus*. The authors attributed this response to the combined effects of Ag^+^ ion release and oxidative stress induced by SeO_2_, which damaged membranes, proteins, and DNA in the target bacteria. Thus, Ag_2_SeO_3_ emerges as a promising next-generation antimicrobial agent for exploration.

Controlling defects in the development of new semiconductors is pivotal for achieving tailored properties. Structural changes at short, medium, and long ranges directly impact local electron density, thereby modulating the reactivity dynamics of the semiconductor.^23^ Typically, these modifications can be engineered during the initial synthesis or through postprocessing treatments. Furthermore, these defects profoundly influence morphology and, consequently, surface reactivity.^24^ This occurs because distinct surface atomic arrangements exhibit varying electron densities, which specifically dictate the production of ROS via the interaction of O_2_ and H_2_O molecules with the semiconductor surface.^25^ Pinatti et al.^26^ successfully modulated defects in Ag_2_SeO_3_ by varying the synthetic approach, employing sonochemical, microwave-assisted hydrothermal, and coprecipitation methods. They observed that the sonochemical route yielded the highest photocatalytic efficiency, attributed to the stabilization of the (001) surface, featuring [AgO_4_·2 V_O_], [AgO_5_·V_O_], and [AgO_6_] surface clusters. Additionally, adjusting the pH during synthesis emerges as a straightforward yet effective strategy for defect control, as it directly influences the nucleation and growth kinetics of the material.^12^

Under irradiation, Ag-based materials—i.e., Ag_2_SeO_3_—may generate metallic Ag nanoparticles exhibit localized surface plasmon resonance, which generated intense, highly localized electromagnetic fields at the metal–semiconductor interface and increases the effective light-absorption cross section, optimizing charge mobility, thereby producing a synergistic effect driven by broadened spectral harvesting, accelerated interfacial charge separation via Schottky-type electron sinks, and enhanced formation of reactive oxygen species (ROS) through optimized O_2_ reduction.^27–29^ In addition to acting as optical antennas, plasmonic Ag domains can serve as active catalytic sites, directly participating in interfacial redox reactions. Our group has previously showed—for Ag/α-Ag_2_WO_4_ and Ag/β-Ag_2_MoO_4_ systems—the incorporation of dispersed plasmonic Ag significantly improves photoreactivity and extends functionality toward antimicrobial and optoelectronic applications.^30,31^ Additionally, the coexistence of Ag^+^/Ag^0^ provides a dual antibacterial action—short-range, highly oxidative surface processes mediated by ROS and longer-range ionic toxicity from Ag^+^ release—so that plasmonic enhancement of photocatalysis often translates into an amplified bactericidal response under illumination and an additive (dark-active) ionic effect in the absence of light.^32^ Recent studies show that for the Ag_2_SeO_3_ system, performance depends critically on morphology, defect density, but also on the formation of composites or heterostructures—such as, Ag_2_SeO_3_/Ppy nanophotocatalyst, Ag_2_SeO_3_/Ag_3_PO_4_/MWCNT/PVDF, etc.—which have already been explored in the literature for organic dyes’ degradation.^26,33,34^

In particular, approaches that produce in situ Ag^0^ (either deliberately as cocatalyst domains or inadvertently via reductive synthesis conditions) can benefit from plasmonic effect leading to optimized response—e.g., faster pollutant removal, stronger ROS generation, and enhanced bactericidal behavior.^29^ In this work Ag_2_SeO_3_ micrometric particles were synthesized under different pH conditions (pH = 2, 5, and 12) using the sonochemical method. This study focuses on elucidating the relationships among synthetic parameters, structural defects, and particle morphology with photocatalytic and bactericidal performance, providing new insights into the development of efficient silver–selenium-based antimicrobial nanomaterials.

## 2 Materials and Methods

### 2.1 Synthesis

Ag_2_SeO_3_ particles were prepared based on the work of Moreno et al.^12^ using 2 × 10^–2^ mol AgNO_3_ (Synth, 99,0%) and 1 × 10^–2^ mol SeO_2_ (Alfa Aesar, 99,4%) as starting reagents. In order to control the pH conditions, 1 M aqueous NaOH (Êxodo científica, 97%) solution was used. First, AgNO_3_ and SeO_2_ were separately dissolved in water −25 and 50 mL, respectively—for 15 min, after which Ag^+^_(aq)_ was added dropwise to the SeO_2_ solution, forming a white precipitate instantaneously. The pH measured for at starting conditions is 2 and was defined as the sample ASOpH2. To obtain samples ASOpH5 (pH = 5) and ASOpH12 (pH = 12), pH was increased by adding NaOH_(aq)_ (1 M)—∼4 and 10 mL, respectively. Then, the suspension was sonicated—without temperature control—for 1 h in a Branson (model 1510) ultrasonic bath with a 120 W (42 kHz, 50 A) input, providing approximately ∼20–80 W to the fluid. The obtained powder was rinsed (5 times) and dried in an oven (60 °C) overnight. The experimental details considered for the synthesis of each of the samples are summarized in Table S1. No uncommon hazards are noted.

### 2.2 Characterizations

The short, medium and long-range structure of the materials were characterized combining Raman (Witec-ALPHA-300R spectrometer (λ_source_ = 633 nm), photoluminescence (PL)—using an Andor (model Kymera) 19.3 cm spectrometer with a 10 mW/355 nm excitation source laser equipped with silicon CCD (Andor Indus)—and UV–visible (UV–vis) spectroscopies—performed in a Shimadzu UV-1800 spectrophotometer (Japan) in diffuse reflectance mode -and X-ray diffraction (XRD) (Rigaku SmartLab SE- Cu K_α_, λ = 1.5406 Å) diffractometer. Raman spectroscopy was conducted over the 100–1000 cm^–1^ range. The PL spectra (380–1200 nm) were deconvoluted based on a Gaussian function. To obtain the optical band gap energy (*E*_gap_), the Kubelka–Munk equation and Tauc plots were used. XRD patterns were indexed using ICSD (inorganic crystal structure database) standards. Particle morphology was assessed by field emission scanning gun electron microscopy (FEG-SEM) operated at 5 kV (Supra 35-VP), and particle size distribution was determined using the software ImageJ.

Finally, X-ray photoelectron spectroscopy (XPS) was performed on a Cienta-Omicron ESCA + equipped with a high-performance hemispheric analyzer (EA 125), with a monochromatic Al K_α_ X-ray source (300 W, hν = 1486.6 eV). The XPS data were analyzed in CasaXPS (version 2.3.27PR4.4) using a Shirley-type background and Scofield cross sections. All data were corrected to the C 1s peak for adventitious carbon, taken to be 284.8 eV, and the suitability of this correction was checked against the core-level binding energies. O 1s, Ag 3d, and Se 3p peaks were fitted using the LA Voigt function in CasaXPS Software.

### 2.3 Photocatalytic Response

Initially, the materials were used to degrade RhB (Synth, 99%). Their photocatalytic response was evaluated under ultraviolet (UV–C) irradiation with six lamps (Philips TUV, 15 W) with a 254 nm dominant wavelength, delivering a light intensity of 1.7 mW cm^–2^ at the reactor surface (20 cm), as measured using a Solar Hukseflux radiometer. The catalytic system was maintained at 25 °C. The photocatalytic reaction took place in a water-cooled glass reactor illuminated from the top. The dispersion was continuously stirred using a magnetic stirrer during the whole process. For each assay, 50 mg of the photocatalyst was suspended in 50 mL of a 1 × 10^–5^ mol/L RhB solution using an ultrasonic bath (Branson, model 1510) for 5 min, followed by stirring in the dark for 20 min to reach adsorption–desorption equilibrium. Aliquots were collected in the beginning in the dark (*t* = −20 min), then when the lights were switched on (*t* = 0 min). The suspension was exposed to UV light under constant stirring under a controlled temperature of 20 °C, and aliquots were collected at specific times (*t* = 5, 10, 15, 30, 60, and 90 min), centrifuged to remove the supernatant, and monitored using a UV–vis spectrophotometer (V-660, JASCO). Analogously to RhB degradation, 50 mg of the photocatalyst was suspended in 50 mL of a 1 ppm of CIP solution using an ultrasonic bath (Branson, model 1510; frequency 42 kHz) for 5 min, then stirred in the dark for 30 min to achieve adsorption–desorption equilibrium. Aliquots were collected when the lights were switched on (*t* = 0 min). The suspension was exposed light under constant stirring under a controlled temperature of 20 °C and aliquots were collected at specific times (*t* = 5, 10, 15, 20, 25, 30, 45, 60, 90, and 120 min), centrifuged to remove the supernatant, filtered using a syringe and 0.45 μm filters, then monitored using high-performance liquid chromatography (HPLC) carried out in an Agilent Infinity II liquid chromatograph equipped with a C18 column. CIP degradation was evaluated using a 75:25 (formic acid: methanol) mixture and monitoring the chromatographic peak yield at ∼4.5 min retention time. Recyclability tests were conducted in order to evaluate the integrity/performance of the photocatalysts over time. To do so the photocatalytic process was repeated over 5 cycles. Between cycles, the photocatalyst powder was recovered and dried.

ROS generation was studied using scavengers’ tests in RhB to evaluate the contribution of e^–^ (silver nitrate—Sigma-Aldrich, 99%), h^+^ (ammonium oxalate—Merck, 99.5%), ^•^OH (*tert*-butyl alcohol—Alfa Aesar, 99+ %), ^•^O_2_H (*p*-benzoquinone—Alfa Aesar, 98+ %), and ascorbic acid (Neon, 99%) in an aqueous medium. Scavengers targets were adopted based on previous works by our group.^15,35^ Additionally, blank the scavengers’ tests were compared to negative controls—performed only in the presence of the photocatalyst. Aliquots were withdrawn at specific times, centrifuged, and analyzed via absorption spectroscopy using a spectrophotometer (Femto Cirrus 80PR). Additionally, probing experiments were carried out using a 1 × 10^–4^ mol/L 9, 10-dimethylanthracene (DMA) (Sigma-Aldrich, 98%) solution (3:2, acetonitrile to water ratio)—singlet oxygen (^1^O_2_) species—and a 1000 ppm coumarin (Sigma-Aldrich, 99%) aqueous solution—^•^OH radicals—to directly evaluate these ROS’ formation. DMA was conducted using a UV–vis spectrophotometer (Jasco, Japan) in the 300–450 nm range, and coumarin in a spectrofluorophotometer (RF-5301 PC, Shimadzu, Japan) in the 250–350 nm range, both taking aliquots at specific times (0, 5, 10, 15, 20, 25, 30, 45, 60, 90, and 120 min). Ag^+^ leaching was quantified using a high-resolution molecular absorption spectrometer (HR-CS MAS ContrAA model 300, Analytik Jena, Jena, Germany, line at 328 nm). For each of the samples—ASOpH2, ASOpH5 and ASOpH12—the leaching experiments were performed using the remaining liquid residue obtained after the photocatalytic process. The filtered liquid was evaluated at room temperature without controlling pH in this solution. The figures of merit were linear range: 0.1–5.0 mg L-1, *R* ^2^ = 0.999, limits of quantification and detection of 0.417 and 0.125 mg L-1, respectively.

### 2.4 Toxicity and Phytotoxicity Assays

To analyze antibiotic efficiency after the bacterial solutions reach a concentration of 1.0 × 10^7^ CFU/mL, a sterile swab will be applied across the entire surface of Mueller–Hinton agar plates. A 4 mm diameter sample disc was placed on top of the cultures, and 10 μL of the CIP solution before and after the photocatalysis was added, ensuring direct interaction with the agar and the microbial culture. After incubating the samples at 37 °C for 24 h, the inhibition zones around the discs were evaluated. All tests were performed in quintuplicate.

For the phytotoxicity evaluation, 20 *Lactuca sativa* seeds were evenly distributed in Petri dishes lined with filter paper, followed by the addition of 4 mL of the liquid residue collected after photocatalysis. Deionized water served as the negative control, while the untreated CIP solution (10 ppm) was used as the positive reference. In the case of sample ASO2C, the treated solution was centrifuged in the presence of NaCl prior to seed exposure to eliminate any leached Ag^+^ ions. All assays were performed in accordance with the protocol reported by Assis et al.^11^. After incubation in the absence of light for 120 h (5 days), the resulting seedlings were analyzed to determine germination percentage and shoot length. Subsequently, the germination index (GI) and relative growth index (RGI) were calculated using eqs 1 and 2.

1 $$GI\;\left(\%\right)=\frac{RLS\times NGS}{RLC\times NGC}\times 100$$

2 $$RGI\;\left(\%\right)=\frac{RLS}{RLC}\times 100$$

where RLS and RLC represent the relative length of the sample and control, respectively, and NGS and NGC the number of germinated seeds in the sample and control.

### 2.5 Antibacterial Assays

The antimicrobial potential of the samples was assessed against *E. coli* (ATCC 25922) and *S. aureus* (ATCC 29213) using the broth microdilution method to establish the Minimum Inhibitory Concentration (MIC). To prepare the inoculum, overnight colonies grown on Mueller–Hinton agar were suspended in 0.9% saline. The suspension’s turbidity was standardized to a 0.5 McFarland scale 1.5 × 10^8^ CFU/mL) via spectrophotometric monitoring at 620 nm, followed by a 1:10 dilution in sterile saline to reach a working concentration of approximately 1.0 × 10^7^ CFU/mL. Assays were conducted in sterile 96-well microplates, initially filled with 100 μL of Mueller–Hinton broth. After performing serial dilutions (2000–1.9 μg/mL) of the samples across the plate to achieve the target concentrations, 100 μL of the bacterial suspension was inoculated into each well. Each plate included positive growth controls (inoculum + medium) and sterility controls (sample + medium). Following a 24 h incubation at 37 °C, 20 μL of a 0.01% (w/v) resazurin solution (Aldrich) was added to each well as a metabolic indicator. After 4 h of incubation, the MIC was determined as the lowest concentration that prevented the colorimetric shift from blue to pink. For the minimal bactericidal concentration, the samples were incubated on Mueller–Hinton agar plates, and the concentration that did not promote bacterial growth was defined as the MBC. All procedures were performed in triplicate to ensure reproducibility.

### 2.6 Metabolic Activity and Intracellular Redox

L929 murine fibroblasts were cultivated in DMEM (VitroCell) supplemented with 10% heat-inactivated fetal bovine serum (FBS, VitroCell), following OECD good in vitro method practices.^36^ cultures were maintained at 37 °C under a 5% CO_2_ atmosphere until reaching 80% confluence. For cytotoxicity assays, cells were exposed to samples via direct contact at concentrations ranging from 1.9 to 7.9 μg/mL for 24 h. Mitochondrial integrity was assessed via the MTT (3-(4,5-dimethylthiazol-2-yl)-2,5-diphenyltetrazolium bromide, Aldrich) colorimetric assay, compliant with ISO 10993-5:2009.^37^ L929 cells were seeded in 96-well plates at 1.0 × 10^4^ and allowed to adhere for 24 h. Following sample exposure (24 h), wells were washed with phosphate-buffered saline (PBS, VitroCell) and incubated with 50 μL of MTT solution (0.5 mg/mL) for 4 h. The resulting formazan crystals were dissolved in 100 μL of isopropanol after the reagent was removed. Absorbance was recorded at 570 nm (BioTek Spectrophotometer). All experiments were conducted in triplicate on three independent occasions (*n* = 9).

Intracellular ROS levels were quantified using the DCFH-DA fluorescent probe (Aldrich).3 Cells were seeded in black 96-well plates and treated with samples at optimal performance concentrations (1.9–7.9 μg/mL). Postexposure, cells were washed twice with PBS and incubated with 100 μM DCFH-DA for 30 min at 37 °C, shielded from light. After a final PBS wash, fluorescence was measured at 485/530 nm (excitation/emission). To evaluate intracellular production of reactive nitrogen species (RNS), nitrite accumulation in the supernatant was measured using the Griess reaction. Briefly, 50 μL of culture supernatant was reacted with 50 μL of Griess reagent (1:1 mixture of 1% sulfanilamide (Synth) in 5% of phosphoric acid (Alfa Aesar) and 0.1% *N*-(1-naphthyl)­ethylenediamine dihydrochloride (Synth)) for 15 min at room temperature. Absorbance was measured at 540 nm. Nitrite concentrations were determined using a standard curve (nM) according to the Sigma-Aldrich G4410 protocol. Both ROS and RNS assays were conducted in triplicate over three independent occasions (*n* = 9).

The statistical analyses were carried out using GraphPad Prism 9.0. The results were expressed as mean ± standard deviation. Differences between groups were assessed using one-way ANOVA followed by Tukey’s post hoc test, and statistical significance was set at *p* ≤ 0.05.

## 3 Results and Discussion

### 3.1 Characterizations

Figure 1a presents XRD patterns of the samples synthesized at different pH conditions. The results confirm the formation of the monoclinic Ag_2_SeO_3_ phase (*P*2_1_/*c*, ICSD n. 78388) in all samples. However, the sample ASOpH12 showed metallic silver (Ag, ICSD n. 893722) as a secondary phase (indicated by the red asterisks), absent in ASOpH2 and ASOpH5, suggesting that alkaline conditions promote partial reduction of Ag^+^ during the sonochemical process. Notably, the intensity of the (032) peak at ∼36.7° decreases significantly as the pH of the solution increases (see Figure 1b). On the other hand, increasing the pH to 12 promotes the (040) diffraction peak at ∼34.7°, indicating a shift in preferred orientation (see Figure 1b). These variations correlate with slower nucleation kinetics under acidic conditions, ASOpH2, resulting in a larger crystallite size (61.74 nm) compared to the samples synthesized in higher pH solutions −5 (55.26 nm) and 12 (52.09 nm).^38^

**Figure 1:**
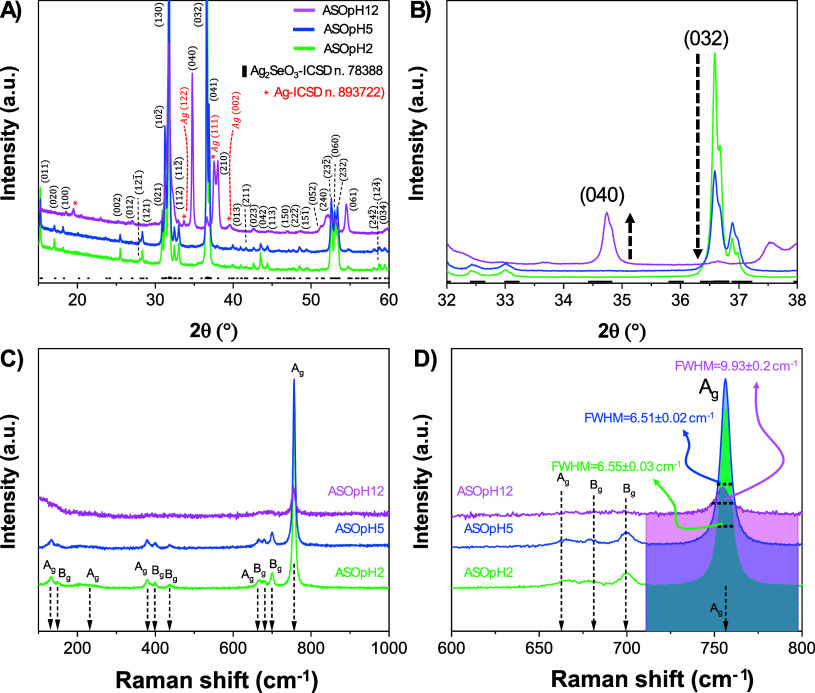
—(A) XRD patterns with a (B) zoomed-in view of the peaks associated with the planes (040) and (032). (C) Raman spectra of Ag_2_SeO_3_ samples synthesized at different pH values (pH = 2, 5, and 12). (D) Zoomed image of the Raman spectra highlighting the changes in specific vibrational modes between (600 and 800 cm^–1^) along with the fwhm values obtained for the Ag mode for samples ASOpH2, ASOpH5, and ASOpH12.

An analysis of the Raman spectra obtained for the samples at room temperature and a 633 nm source laser (Figure 1c) further supports the XRD data. All samples show characteristic vibrational modes of the Ag_2_SeO_3_ symmetry.^12,26^ At ∼750 cm^–1^, the Ag mode is characteristic of the SeO_3_^2–^ stretching. In ASOpH12, this mode exhibits a broader full width at half-maximum (fwhm = 9.93 ± 0.2 cm^–1^) compared to ASOpH5 (6.5 ± 0.03 cm^–1^) and ASOpH2 (6.1 ± 0.02 cm^–1^), as shown in Figure 1d. This broadening suggests an increase in local disorder and possibly a higher concentration of structural defects or lattice strain, which may be associated with the reduction of Ag and phase separation. The appearance of metallic Ag and the vibrational broadening at pH 12 may influence the material’s surface chemistry and electron transfer dynamics, which are critical to its antimicrobial performance. In contrast, samples synthesized at lower pH maintain a purer Ag_2_SeO_3_ phase with more defined vibrational signatures, indicative of greater structural uniformity. Furthermore, ASOpH12 shows suppression of some Raman modes, which suggests lower short-range order in these particles.

The XPS spectra of the Ag 3d region (Figure 2a–c) display the characteristic doublet corresponding to Ag 3d_5/2_ and Ag 3d_3/2_, separated by a spin–orbit splitting of approximately 6.0 eV. Deconvolution of the peaks reveals the coexistence of Ag in multiple oxidation states, including Ag^0^ and Ag^+^.^39^ The relative proportions vary among the samples, with Ag^+^_L_ (Ag–O/Ag–Se lattice Ag^+^) being the predominant species in all cases (75.7%, 68.7%, and 65.3% in samples ASOpH2, ASOpH5, and ASOpH12, respectively). Additionally, a defective Ag^+^ (Ag^+^_D_) state is observed in all samples, and its percentage increases with increasing synthetic pH relative to ASOpH2 (21.2%).^40,41^ On the other hand, Ag^0^ appears at lower levels in all samples, but at the highest level in sample ASOpH12. Notably, the higher the Ag^+^_D_ percentage, the higher the Ag^0^ levels. The persistence of metallic Ag^0^ likely arises from partial reduction during synthesis and may be beneficial to the photocatalytic response, which can be ascribed to the optimization of charge separation efficiency and the plasmonic effect.^26,42,43^

**Figure 2:**
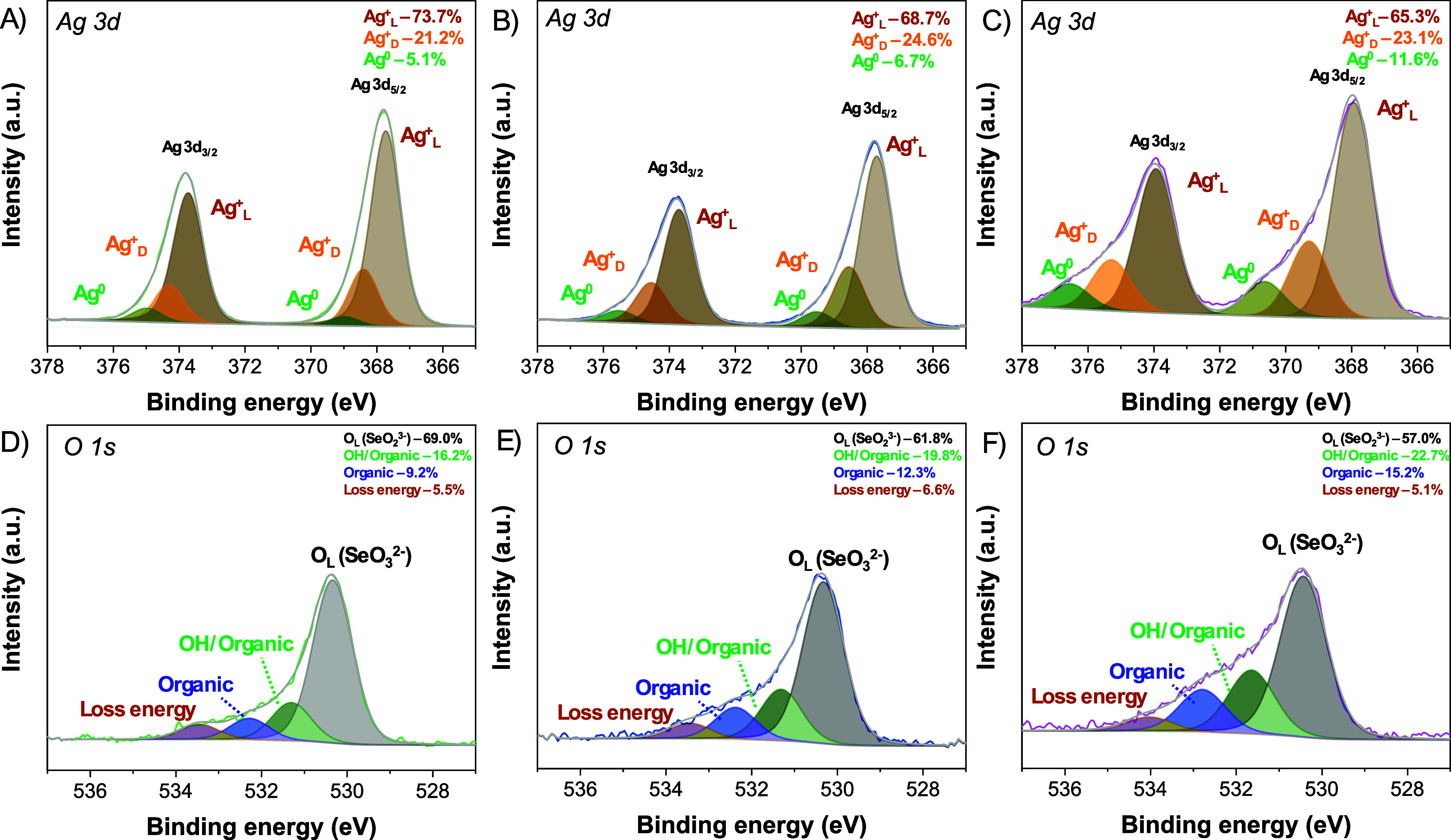
—Ag 3d and O 1s high-resolution XPS spectra obtained for the samples (A,D) ASOpH2, (B,E) ASOpH5 and (C,F) ASOpH12.

Complementary insights are provided by the O 1s spectra (Figure 2d–f), which can be deconvoluted into contributions from lattice O (O_L_, [AgO_6_] and [SeO_3_] clusters), surface OH/organic O species and adsorbed O species along with a minor contribution from energy loss features.^44,45^ The dominance of lattice O confirms the structural integrity of the Ag–O and Se–O frameworks. However, its percentage decreases significantly in sample ASOpH12, suggesting that alkaline media hinders the stability of the Ag_2_SeO_3_ lattice, which may result in more structural defects.^46^ On the other hand, the presence of OH can be associated with the higher concentration of NaOH used for higher pHs. These leftover surface terminations may improve the generation of ROS, such as ^•^OH radicals under illumination. Additionally, one may also consider that higher pH may produce surface functionalization to some extent, for instance packing ASOpH12 surface with OH terminations, which may readily generate ^•^OH radicals, enhancing photocatalytic performance. A slight change in the Se 3p spectrum for each of the samples—Figure S1­(a–c)—signals that the pH conditions influence coordination of the Se ions as the pH increases. This may be ascribed to the reduction of the silver ions within the Ag_2_SeO_3_ lattice to form metallic silver (Figure S2), which directly affect net formation—Se—order.

Pinatti et al.^26^ reported that plate-like and rod-like Ag_2_SeO_3_ morphologies originate from preferential growth along crystallographic directions associated with relatively low surface energies, particularly the {001}, {011}, and {111} families. Intermediate metastable morphologies evolve toward more compact or faceted structures as the system minimizes its total surface energy. Figure 3a–c reveals that samples ASOpH2 exhibit well-defined microrod particles, with a wide size distribution (X̅ = 4.5 ± 2.3 μm), with elongated, face-rectangular morphologies, more prominent under acidic conditions and which are indicative of anisotropic growth along low-energy facets such as {001} and {011}. Increasing pH to a slightly acidic condition (ASOpH5) partially suppresses this oriented particle growth, resulting in shorter, beveled, microrods (X̅ = 1.6 ± 0.9 μm). Finally, synthesis in an alkaline medium completely impairs rod-like particle formation, favoring monodispersed sheet-like particles (X̅ = 0.9 ± 0.3 μm), suggesting pronounced surface reconstruction and stabilization of higher-energy facets. These results are well aligned with the XRD analysis, which indicates the emergence of a diffraction peak ascribed to the plane (040) in sample ASOpH12. This interpretation suggests that alkaline conditions modify relative surface energies—likely via OH^–^ adsorption—stabilizing nonconventional planes and disrupting preferential elongation pathways. Consequently, crystal growth follows an alternative energetic route dominated by surface reconstruction rather than axial extension, favoring compact, multifaceted morphologies with enhanced surface exposure.

**Figure 3:**
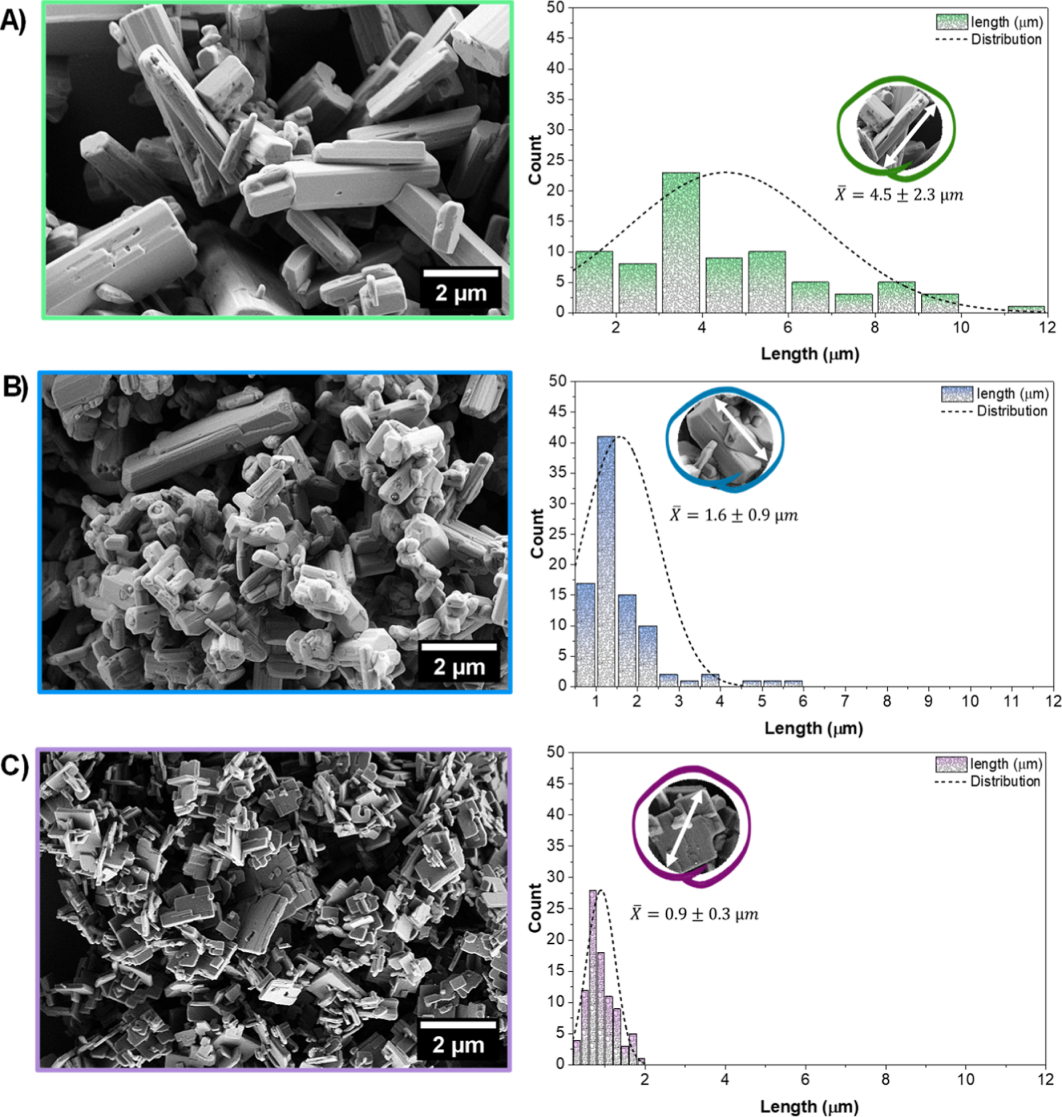
FE-SEM micrograph images indicating changes in particle size and morphology for all samples synthesized in pH = (A) 2, (B) 5, and (C) 12. *particle size for each of the samples was measured for ∼90 particles, selected from 2 different regions’ micrograph images (10k magnification).

Thus, the pH of the reaction medium plays a key role in shaping Ag_2_SeO_3_ morphology by modulating nucleation, growth, and precursor chemistry under sonochemical conditions. An acidic medium accelerates nucleation and favors microrod formation via facet-selective adsorption. Increasing pH promotes anisotropic growth, yielding shorter rods.^47,48^ Further base addition completely suppresses rod growth. pH also influences precursor solubility and cavitation dynamics, collectively explaining the morphological differences observed.

The PL spectra (Figure 4a–d) provide insight into the electronic structure of the Ag_2_SeO_3_ system in the different pH conditions. Figure 4a shows a comparison of the PL spectrum obtained for each of the samples. A PL emission peak extending from ∼550 to ∼900 nm—with maximum intensity at ∼690–700 nm—is characteristic of the Ag_2_SeO_3_ electronic structure, which is related to the [AgO_6_] and [SeO_3_] clusters that compose its lattice. The results show a clear reduction in intensity as the synthesis’s pH increases. Quenching of the PL signals can be attributed to changes in charge-carrier dynamics, indicating more optimized electron segregation and prolonged ROS generation resulting from a more controlled electronic decay.^49^ Overall, the PL data demonstrates that synthesis pH strongly influences the type and distribution of defects in Ag_2_SeO_3_.

**Figure 4:**
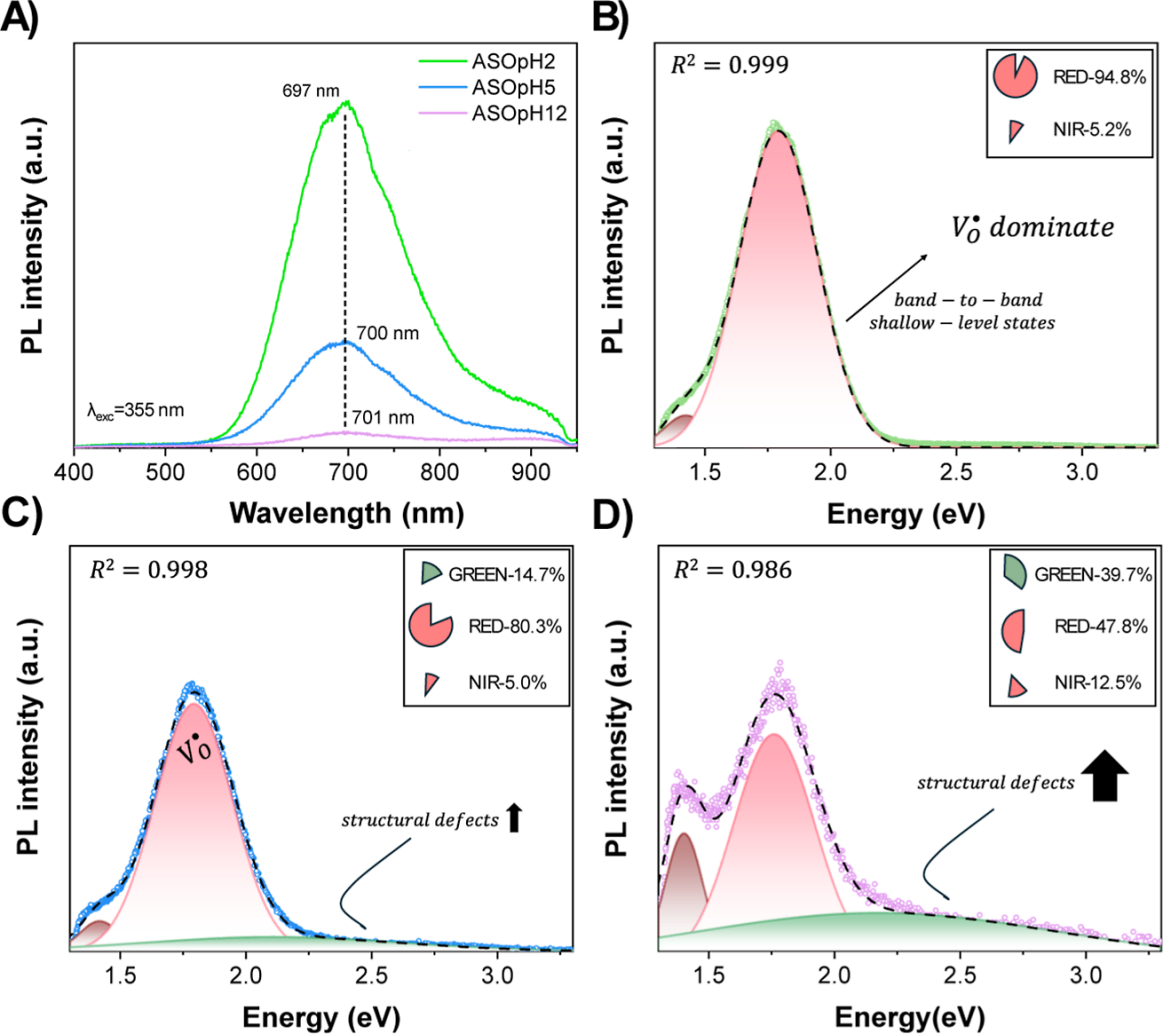
—(A) shows a comparison between the different PL spectra excited in the UV-region (λ_exc_ = 355 nm), and the deconvoluted PL spectra is shown for samples (B) ASOpH2, (C) ASOpH5, and (D) ASOpH12.

While acidic conditions induce phase segregation and enhance oxygen-related vacancies (*V*_O_), basic media promote structural defects. These variations in the electronic defect structure are expected to impact charge carrier dynamics, surface reactivity, and ultimately the antimicrobial and photocatalytic behavior of the material.^12,50^ ASOpH2 exhibits a broad, red-shifted emission centered near 695 nm (Figure 4b), dominated by deep-level states, indicated by its large red emission (>90%). These results may be associated with structural defects due to the presence of distortions on the crystalline lattice of Ag_2_SeO_3_. Thus, it can be inferred that acidic pH favors the formation of intrinsic defects during synthesis. At pH 5 (ASOpH5, Figure 5c), there is a blue shift and increased contribution from higher-energy states, which can be associated with shallow-level defects within the band gap region, and the presence of *V*_O_. Hence, changing the pH from 2 (acidic) to a slightly acidic environment (pH = 5) changes, displaying slightly more energetic, structure-related defect types—green emission peak. For ASOpH12 (Figure 5d), the PL spectrum becomes broader and more complex, with a significant shift toward green emissions (∼2.4 eV) apart from the red emission peak (∼1.8 eV). The emergence of Ag^0^ in this sample, confirmed by XRD and XPS, likely introduces additional nonradiative recombination centers and surface plasmon effects, altering the defect recombination pathways.^51^ The shift in emission peak to ∼700 nm (Figure 5d)) and the presence of intense midgap states support the generation of *V*_O_ and disorder-driven trap states.^52^

**Figure 5:**
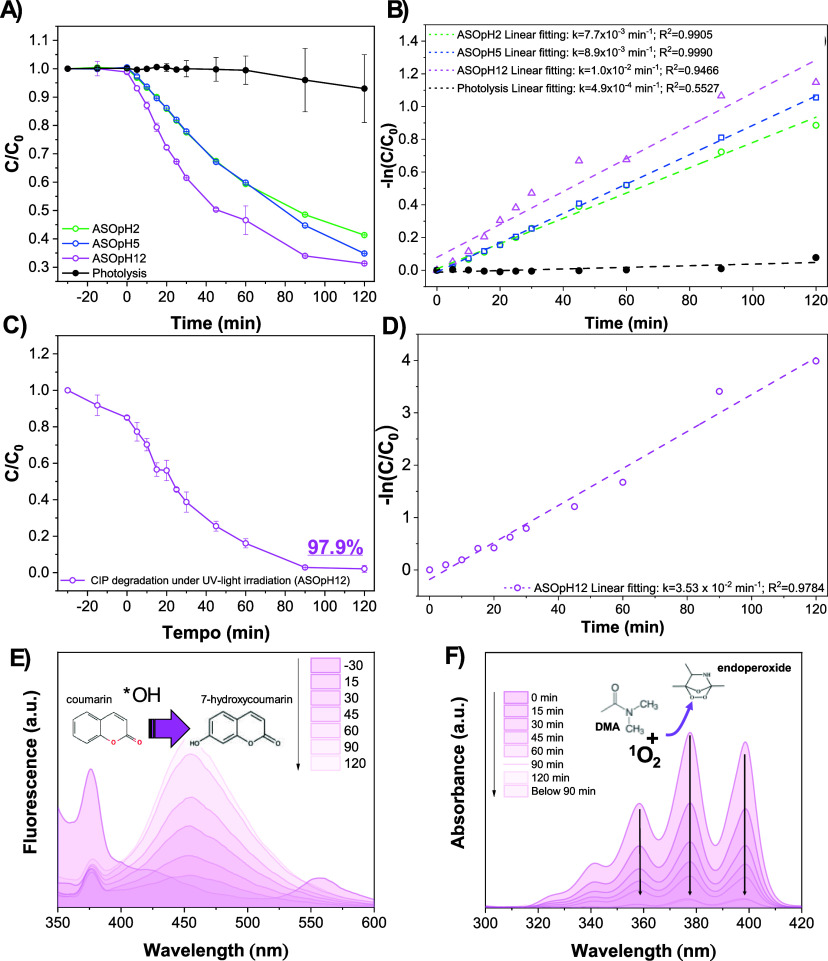
—CIP photocatalytic (A) efficiency and (B) kinetics measured for all samples. (C,D) show the degradation efficiency and kinetics using HPLC of sample ASOpH12, which behaved the best compared to samples ASOpH2 and ASOpH5. (E) and (F) display probing experiments using coumarin—to detect ^•^OH—and DMA—to detect ^1^O_2_—respectively. All measurements were performed in duplicates with the standard errors reported as error bars.

The XRD and XPS analyses confirmed the presence of metallic Ag^0^ in this sample. Thus, the pronounced PL quenching and spectral redistribution observed for ASOpH12 cannot be attributed solely to defect formation but also suggest localized surface plasmon resonance effects. The Ag^0^/Ag_2_SeO_3_ interface likely acts as an efficient electron sink, promoting interfacial charge transfer and suppressing radiative recombination, as evidenced by the marked reduction in PL intensity. Furthermore, plasmon-induced local electromagnetic field enhancement may modify defect-state occupation and recombination pathways, leading to the increased contribution of intermediate (green) emission bands. Therefore, the improved charge separation inferred from PL results may be ascribed to a synergistic combination of defect engineering and plasmonic effects, which directly correlates with the superior photocatalytic activity of ASOpH12.

### 3.2 Photocatalytic Response

The photocatalytic performance of each of the samples is displayed in Figure 5a–d. The results show a clear dependence on the synthesis pH, with ASOpH12 exhibiting the most efficient and kinetically favorable response. Figure 5a shows the performance of the photocatalysts over time using UV–vis spectroscopy. While photolysis alone results in negligible degradation over 120 min (*C*/*C*_0_ ≈ 0.95), all ASO-based samples promote a marked decrease in pollutant concentration, confirming their photocatalytic activity. Among them, ASOpH12 displays the fastest degradation rate, reaching ∼60–65% removal within 120 min with sample ASOpH12 outperforming ASOpH2 and ASOpH5. The pseudo-first-order kinetic analysis (Figure 5b) further quantifies this trend. The apparent rate constants (*k*) increase systematically with pH, following the order: ASOpH12 (*k* = 1.0 × 10^–2^ min^–1^)≫ASOpH5 (*k* = 8.9 × 10^–3^ min^–1^)>ASOpH2 (*k* = 7.7 × 10^–3^ min^–1^)≫photolysis (*k* = 4.9 × 10^–4^ min^–1^). *k* for sample ASOpH12 is approximately 20 times higher than photolysis, evidencing the strong synergistic role of the catalyst in accelerating the degradation pathway. Although UV–vis is valuable for screening and comparative analysis, HPLC offers a more accurate and mechanistically meaningful assessment of photocatalytic efficiency.^53^ Furthermore, the ln­(*C*_0_/*C*) plots for all Ag_2_SeO_3_ samples exhibited excellent linearity over the entire 120 min irradiation period (*R* ^2^ > 0.94), indicating stable apparent rate constants without evidence of diffusion limitations or catalyst deactivation. Conversely, photolysis showed poor linearity (*R* ^2^ ≈ 0.55), confirming that direct UV degradation does not follow pseudo-first-order kinetics and reinforcing that the catalytic surface establishes the controlled kinetic regime.

The behavior observed in Figure 5c,d—resolved chromatographically by HPLC—indicates near-complete removal of CIP (∼98%) over 120 min, along with the higher and more reliable apparent rate constant (*k* = 3.53 × 10^–2^ min^–1^). This enhanced performance under basic conditions can be ascribed to different factors including: 1) the morphological transition from microrods to smaller, sheet-like particles, which facilitates charge transfer; 2) a slight reduction in medium-range order, favoring localized states that enhance visible-light absorption, and the presence of oxygen-related defects that act as charge-trapping sites, suppressing electron–hole recombination^3,54^ ) XPS and XRD analyses confirm the coexistence of Ag^0^ and Ag^+^ species and minor Ag peaks, suggesting that mixed-valence silver and surface plasmon effects further enhance charge separation; (4) OH surface functionalization to optimize ROS generation. Altogether, the synergistic combination of morphological refinement, *V*_O_, and mixed Ag valence states accounts for the superior photocatalytic response of ASOpH12. Furthermore, recyclability tests (Figure S4) indicate that the efficiency of sample ASOpH12 remains stable over five cycles with no significant performance loss.

Figure 5e,f display the results obtained in probing experiments, conducted in coumarin and DMA. The data provides significant information on the dynamics of ROS generation, more specifically regarding ^•^OH radicals and ^1^O_2_ species. The fluorescence spectra (Figure 5e) display an increase in the intensity of the peak ascribed to 7-hydroxycoumarin, which indicates that in an aqueous medium, the semiconductor promotes the generation of ^•^OH radicals at a rate that increases with time, as ^•^OH and coumarin combine to form 7-hydroxycoumarin molecules. Analogously, DMA probes indicate the formation of ^1^O_2_ species, inferred from the observed quenching of the DMA absorbance signal, as it is degraded by ^1^O_2_. Coumarin and DMA probing can be correlated with scavenger experiments to deepen our understanding of the mechanisms controlling the photocatalytic degradation of CIP molecules.^55^ The scavengers’ tests—conducted for sample ASOpH12, which outperformed the other samples—are displayed in Table 1, identifying the dominant reactive species responsible for CIP degradation.

**Table 1:** —Scavengers Tests Performed for Sample ASOpH12 in a CIP Solution

scavenger	scavenged species	degradation (%)
blank	-	98.83
potassium biphtalate	^•^OH	94.72
ascorbic acid	^1^O_2_	66.95
*p*-benzoquinone	^•^O_2_H	25.15
diammonium oxalate	h^+^	95.39
silver nitrate	e^–^	88.69

In these tests, potassium biphthalate (^•^OH scavenger) and diammonium oxalate (h^+^ scavenger) produced A slight decrease in degradation (from 98.8% to 94.7% and 95.4%, respectively), which suggests that ^•^OH generated at the valence band (VB, h^+^) have limited contribution to the overall reaction. Addition of AgNO_3_ (e^‑^ scavenger) produces a modest performance decrease (88.7%), suggesting that free electrons participate but are not the primary oxidants. In contrast, the presence of *p*-benzoquinone (^•^O_2_H scavenger) caused a dramatic suppression of activity (from 98.8% to 25.2%), while ascorbic acid (^1^O_2_ scavenger) also significantly decreased performance (to 67.0%). These results indicate that both ^•^O_2_H radicals and ^1^O_2_ species act complementarily toward the degradation of CIP. Apart from ^1^O_2_, the probing experiments also indicate the ^•^OH radical formation. Scavenger tests provide further insight, suggesting that ^•^OH reacts further in the medium, evolving into ^•^O_2_H radicals, which then drive degradation.

Moreover, this hypothesis is consistent with the XPS data, which reveal the presence of metallic silver (Ag^0^) on the surface of Ag_2_SeO_3_, acting as an electron sink to improve charge transport and segregation, thereby enhancing O_2_ reduction. The reaction steps associated with the generation of ^•^O_2_H radicals and ^1^O_2_ species. To fully understand photocatalyst performance, especially the higher ASOpH12 efficiency, one must consider the plasmonic effect associated with the presence of surface Ag^0^, which enhances charge transfer.^56^ The plasmonic effect arising from the in situ formation of metallic Ag^0^ nanodomains significantly enhances photocatalytic efficiency by generating intense, highly localized electromagnetic fields at the metal–semiconductor interface through localized surface plasmon resonance.^57^ These nanometric field confinements increase the effective absorption cross-section of the system, allowing low-energy photons from the visible and near-infrared regions to be efficiently harnessed, which are otherwise weakly absorbed by pristine Ag_2_SeO_3_. The strong local field gradients optimize electronic transitions and promote multiphoton excitation processes even under low-intensity. Simultaneously, the Ag^0^/Ag_2_SeO_3_ heterointerface acts as an efficient charge-separation platform, where plasmon induces electronic excitation into the conduction band of Ag_2_SeO_3_, thereby suppressing charge recombination. This synergistic combination of enhanced light harvesting, broadened spectral response, and improved charge-carrier dynamics ultimately accelerates interfacial redox reactions, thereby leading to superior photocatalytic activity.^58,59^

Irradiation of the semiconductor with a light source of sufficient energy (i.e., an UV source) initiates a flux of excited electrons^60^ toward the conduction band, producing an electronic density gradient where the conduction band (CB) becomes increasingly negatively charged (δ_CB_^–^), filled with excited CB electrons, leaving the VB positively charged (δ_VB_^+^), dominated by holes (eq 3). O_2_ molecules adsorb and are reduced by e^–^ to generate ^•^O_2_^–^ (eq 4), whereas at the h^+^ hydrolyze H_2_O_(ads)_ producing ^•^OH radicals and protons (H^+^) (eq 5).

3 $${Ag}_{2}SeO_{3}\overset{h\nu }{\to }{Ag}_{2}{SeO}_{3}\left(e_{CB}^{-}+h_{VB}^{+}\right)$$

4 $$O_{2\left(ads\right)}+e_{CB}^{-}\to {\cdot O}_{2}^{-}$$

5 $$H_{2}O_{\left(ads\right)}+h_{VB}^{+}\to \cdot OH+H^{+}$$

Favored by the slightly acidic medium, in which the reaction takes place (pH ∼ 5), the ·O_2_^–^ radicals further react with H^+^ ions to form the ^•^O_2_H radical (eqs 6 and 7), which can also stem from ^•^H_2_O_2_-^•^OH and O_2_–H^+^ reactions (eqs 8 and 9).

6 $${\cdot O}_{2}^{-}+H^{+}\to \cdot O_{2}H$$

7 $$O_{2}+H^{+}\to \cdot O_{2}H$$

8 $$2\cdot OH\to {H_{2}O}_{2}$$

9 $${H_{2}O}_{2}+\cdot OH\to \cdot O_{2}H+H_{2}O$$

Finally, in a secondary alternative pathway, ·O_2_^–^ radicals may react at the δ_VB_^+^, resulting in the ^1^O_2_ (eq 10).

10 ·O2−+hVB+→O21

The data at hand helps understand why, although present in the medium—as evidenced by the coumarin probes—^•^OH radicals does not act directly toward CIP degradation. The full mechanistic pathways are represented in Figure 6a,b, considering ROS generation (Figure 6a) and the plasmonic effect (Figure 6b) in CIP degradation, as well as postphotocatalysis toxicity analysis.

**Figure 6:**
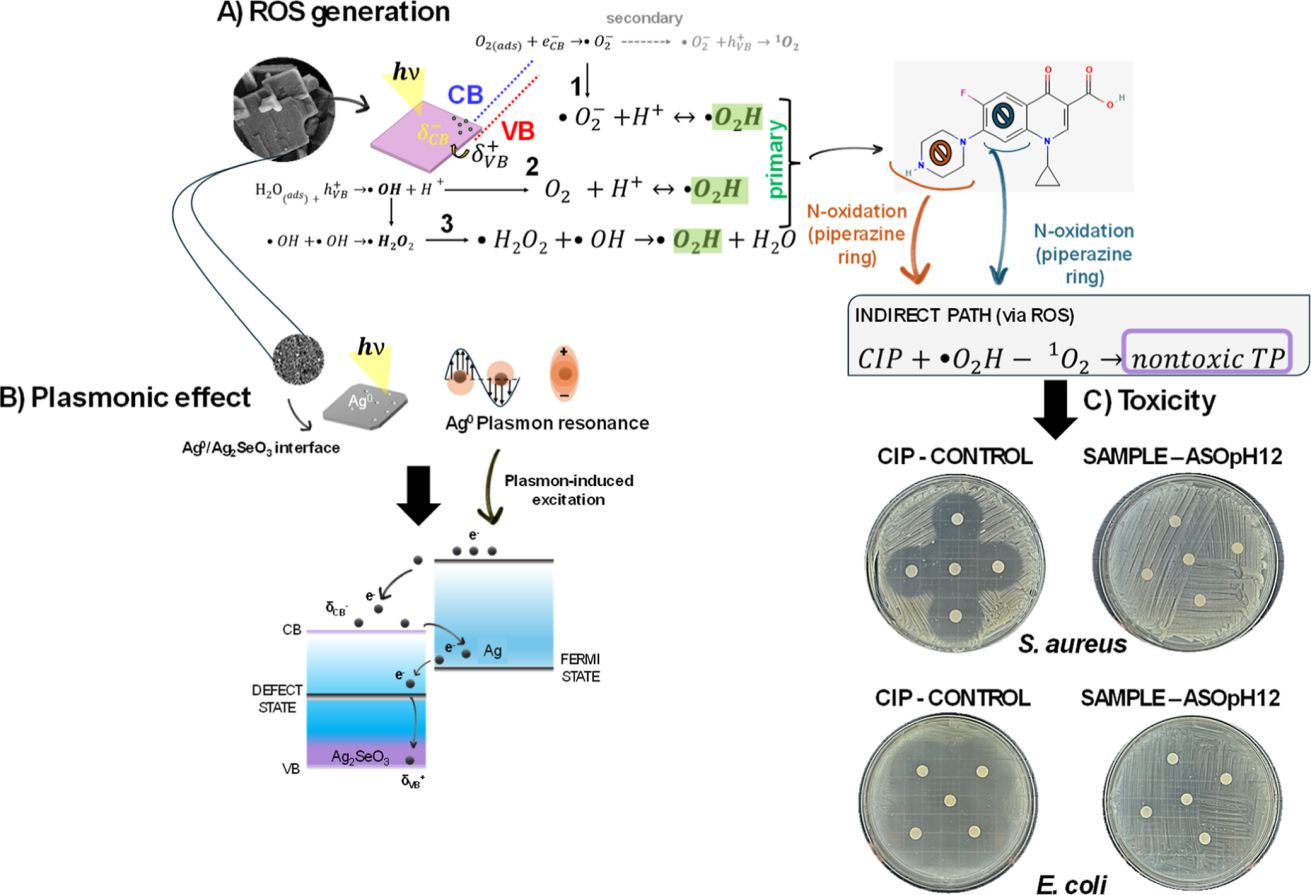
—(A) Proposed mechanistic pathways including ROS species’ generation toward the degradation of the CIP molecule, considering enhanced charge-separation by the (B) plasmonic effect contribution. (C) Shows toxicity assays performed using the sample ASOpH12 photocatalysis’ residue against *S. aureus* relatively to a CIP solution.

Figure 6c shows toxicity assays conducted with the CIP residue toward *S. aureus* and *E. coli* bacteria. Compared to the control (CIP solution)—which proved toxic, as expected—the residue did not impair bacterial growth in neither. The lack of antibacterial activity observed indicates that CIP was degraded/removed from the solution to form nontoxic transformation products. Additionally, *L. sativa* seeds were also used to evaluate the phytotoxicity. The results are summarized in Table 2. The residue showed similar germination rates compared to the control, but longer plantlets were observed, which explains the larger GI and RGI values. Thus, it can be inferred that the samples’ residue shows no toxicity, similarly to the seeds exposed to pure water. Furthermore, a germination rate lower than 100%—observed for the control sample can be ascribed to bad seeds, which can also be the case for the seeds irrigated with the photocatalysis’ residue. Thus, the toxicity and phytotoxicity results suggest that the CIP molecules are, in fact, actively and efficiently degraded into nontoxic transformation products, harmless both to bacteria and the *L. sativa—*which is very sensitive to environmental variations.

**Table 2:** —Phytotoxicity Assays Conducted for *L. sativa* Seeds^a^

sample	lactuca sativa					
	germination (%)	length (cm)	GI (%)	RGI (%)	observations	ref
negative CTRL	80	1.4 (0.8)	-	-	-	this work
positive CTRL	75	1.9 (0.9)	80.3	85.7	atrophiated structure with dark spots in the seeds	
ASOpH12	85	2.0 (0.8)	152	142	germination rate similar to the control, with longer platlets observed	

a GI and RGI (germination and relative growth index, respectively) were calculated—respective to negative (H_2_O) and positive (CIP) controls—based on germination rate and the length measured for the plantlets over 120 h incubation.

The photocatalytic efficiency of the samples in this work was compared with previously reported data on Ag_2_SeO_3_-based materials’ photocatalysis (Table 3). In general, pristine or composite Ag_2_SeO_3_ systems display highly variable activity depending on morphology, cocatalysts, and light source. For instance, simple Ag_2_SeO_3_ composites such as PEDOT/Ag_2_SeO_3_ and Ag_2_SeO_3_/Ag_3_PO_4_/MWCNT/PVDF exhibit relatively low efficiencies, reaching only 23% and 31% degradation under incandescent and LED irradiation, respectively, after prolonged exposure times of 210 and 40 min. Conversely, optimized systems such as Ag–Ag_2_SeO_3_/Ppy and Ag_2_SeO_3_–SC demonstrate significantly improved responses, achieving 82.9% and complete degradation within 25 and 60 min, respectively, under LED or UV light. Notably, the highest efficiencies were observed for hierarchical nanostructures (e.g., ASOpH5), in which 100% RhB removal was obtained in only 30 min under UV irradiation.

**Table 3:** —Performance Comparison of Ag_2_SeO_3_-Based Materials in the Photodegradation of Organic Pollutants^a^

photocatalysts	synthesis	pollutant	efficiency (%)	exposure (min)	source	conditions	ref
pH12	SC	CIP	100				this work
PEDOT/Ag_2_SeO_3_	LLIS	RhB/MB	23	210	Incandescent	15 mg catalysts, 30 mL of ppm RhB, *T* = 35 °C, pH = 7	^61^
Ag_2_SeO_3_/Ag_3_PO_4_/MWCNT/PVDF	CP	IC	31	40	LED	10 mg catalysts, 10 mg/L RhB, pH = 6	^34^
Ag–Ag_2_SeO_3_/Ppy	CP	RhB/MB	82.86	25	LED	20 mg catalyst, 20 mg/L RhB, pH = 8	^62^
Ag_2_SeO_3_	SC, UT, MAH, CP	RhB	100	60	UV–C	50 mg catalyst, 50 mL 1 × 10^–5^ mol/L RhB, *T* = 20 °C	^26^
ASOpH5	SC	RhB	100	30	UV–C	15 mg catalyst, 50 mL 1 × 10^–5^ mol/L RhB	^12^

a SC: sonochemistry; LLIS: liquid–liquid interfacial synthesis; CP: coprecipitation; UT: ultrasonic tip; MAH: microwave-assisted hydrothermal. RhB: rhodamine B, CIP: ciprofloxacin; MB: methylene blue; IC: indigo carmine.

### 3.3 Antibacterial and Cytotoxicity Assays

MIC assays performed against *S. aureus* (Gram-positive) and *E. coli* (Gram-negative) reveal that the antimicrobial activity of the Ag_2_SeO_3_-based samples is strongly dependent on the synthesis pH (Figure 7a,b). For *S. aureus* (Figure 7a), the material synthesized at acidic pH (ASOpH2) exhibited the highest antibacterial efficiency, with a sharp reduction in bacterial viability occurring at concentrations around 15.6–62.5 μg/mL, and complete inhibition above ∼125 μg/mL. The samples synthesized at slightly acidic (ASOpH5) and alkaline conditions (ASOpH12) required higher concentrations to achieve comparable inhibition, reflecting a diminished activity against Gram-positive bacteria (250 μg/mL). For *E. coli* (Figure 7b), a slightly different trend was observed. While all samples demonstrated significant bactericidal action, the ASOpH5 material displayed the steepest curve, achieving total growth suppression at concentrations as low as 62.5 μg/mL. The samples ASOpH12 and ASOpH2 also exhibited inhibitory effects, but complete inhibition required slightly higher concentrations. This suggests that structural or surface chemistry differences induced by the synthesis pH play a role in affinity toward Gram-negative membranes, possibly related to differences in Ag^+^ ion release, particle size, surface reactivity, and ROS production.

**Figure 7:**
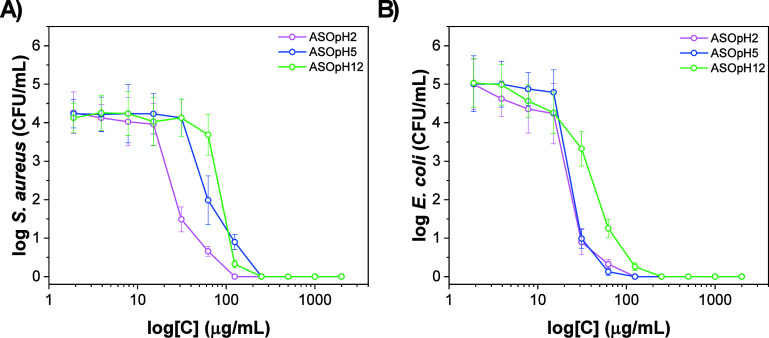
—Antimicrobial activity (MIC) toward (a) *S. Aureus* (gram + ) and (b) *E. coli* (gram – ) bacteria. All experiments were conducted in triplicate on three independent occasions (*n* = 9).

In fact, the results show that Ag^+^ release in the medium vary increase from 1.56 ± 0.05 mg/L (ASOpH2) to 1.82 ± 0.06 mg/L (ASOpH5) to 3.87 ± 0.19 mg/L (ASOpH12). The bactericidal response of the Ag_2_SO_3_ samples can be rationalized by considering both the extent of Ag^+^ ion release and the nature of the ROS generated under light irradiation, while acknowledging that these materials are also able to produce ROS in the absence of light, albeit at lower levels than under illuminated conditions.^61–63^ The Ag^+^ leaching increased progressively with the pH increase, indicating that the material obtained in alkaline conditions promote a higher ionic dissolution of silver. Since Ag^+^ ions can interact with thiol and amine groups in cellular proteins, leading to enzyme inactivation and membrane destabilization, their higher availability in ASOpH12 may enhance bactericidal effects, especially against Gram-negative *E. coli*, whose thinner peptidoglycan layer allows easier ion penetration.^64^

In addition to the ionic contribution, the ROS mechanism is also relevant in bacterial inactivation. Samples ASOpH2 and ASOpH5, which predominantly depend on ^•^OH radicals—primarily—and ^1^O_2_ species, are expected to induce severe oxidative damage to cell walls and membranes through nonselective oxidation of lipids, proteins, and polysaccharides. The ^•^OH radical is among the most reactive ROS and has limited diffusion capability, implying that its effect is mainly surface-driven.^12^ Consequently, *S. aureus* (Gram-positive), which has a thick peptidoglycan layer, may exhibit partial resistance to these short-lived species, while *E. coli* remains more vulnerable due to its relatively permeable outer membrane.^65,66^ In contrast, ASOpH12 mainly produces ^•^O_2_H and ^1^O_2_ species, which possess lower selectivity compared to ^•^OH radicals. Thus, ROS species act complementing the stronger Ag^+^ release in this sample. Therefore, the combined effect of elevated Ag^+^ concentration and diffusible ROS in ASO, independently of synthesis pH, is expected to cause pronounced damage to both bacterial types, though *E. coli* would likely experience faster inactivation due to its less protective cell envelope.^67,68^ The MIC and MBC assays were carried out in an incubator at 37 °C for 24 h under dark conditions, following standard antibacterial testing protocols. Although no light irradiation was applied during the microbiological experiments, ROS-based mechanisms was included since these materials are also able to generate ROS in the absence of light—at lower proportions—but which are still relevant, as reported in the literature. In addition, the structural and surface properties induced by the synthesis pH influence both Ag^+^ release and surface reactivity, which contribute to bacterial inactivation even under dark conditions.

These results demonstrate a selective trend toward Gram-positive (ASOpH2) and Gram negative (ASOpH5/12) bacteria, which indicates that tuning the synthesis conditions can optimize Ag_2_SeO_3_ nanostructures for specific antibacterial targets. Complementary to the MIC results, Table 4 shows the MBC for each of the samples. The results are closely related with MIC data, confirming that sample ASOpH2 was the most effective toward killing the bacteria.

**Table 4:** —MBC towards *S. Aureus* and *E. Coli* Bacteria

sample	S. aureus (μg/mL)	E. coli (μg/mL)
ASOpH2	125	125
ASOpH5	250	125
ASOpH12	250	250

Table 5 provides a comparative overview of the antimicrobial performance of Ag_2_SeO_3_ synthesized in this work under controlled pH conditions compared to other Ag-based materials reported in the literature, highlighting the influence of synthesis route, morphology, and active mechanisms on bactericidal efficacy. The samples in this work exhibit 100% inhibition against both *S. aureus* and *E. coli* at a dose of 125 μg/mL, which is competitive with or superior to many previously reported Ag-based systems, despite their comparatively larger micrometric size and non-nanoscale morphology.

**Table 5:** —Comparison of the Antimicrobial Response and Mechanism Associated with Different Ag-Based Compounds

material	synthesis	size/morphology	test organism (s)	assay	dose (μg. mL^–1^)	result	proposed mechanism	ref
Ag_2_SeO_3_	SC	1–9 μm/rod, sheet like	S. aureus (ATCC 29213)/E. coli (ATCC 25922)	MIC	125	100% (S. aureus)/(E. coli) at 500 μg. mL^–1^	ROS (^1^O_2_ and ^•^OH species)/Ag^+^ leaching	this work
Ag_2_Se	green bacterial biosynthesis	30–40 nm/spherical	S. aureus/E. coli	MIC	250/150	80% at 200 μg. mL^–1^	-	^69^
Ag_2_Se	plant extract (Mlilotus officinalis) green synthesis	20–40 nm/spherical	P. aeruginosa (ATCC 27853)	MIC	6.25	potent inhibition at 100 μg. mL^–^	membrane disruption, ROS, Ag^+^ action	^19^
Se-NP	laser ablation	100 nm/spherical	S. aureus/E. coli	MIC	50	100% at 79 (S. aureus)/107 μg. mL^–1^ (E. coli)	-	^70^
BI/Ag-NPs	chemical reduction	9 nm/quasi-spherical	S. aureus (ATCC 29213)/E. coli (ATCC 11229)	MIC	1.72/3.44	100	-	71

The metabolic activity of L929 cells was assessed via MTT assay across a range of concentrations (Figure 8). Adhering to established by ISO 10993-5:2009, cell viability below 75% was defined as the threshold for potential cytotoxicity. Therefore, the extract at 7.9, 3.9, and 1.9 μg/mL (obtained by serial dilution) were evaluated, since concentrations above 7.9 μg/mL resulted in complete loss of cell viability (0%). At the highest concentration (7.9 μg/mL), all samples fell below this safety limit, with mean viability values of 50.2% (ASOpH2), 38.4% (ASOpH5), and 16.0% (ASOpH12). At the intermediate dose (3.9 μg/mL), ASOpH2 (85.4%) and ASOpH5 (76.5%) remained near or above the safety threshold, whereas ASOpH12 showed significant cellular compromise at 56.0%. Conversely, at the lowest concentration (1.9 μg/mL), all samples were biologically acceptable, exceeding the cytotoxicity limit with values ranging from 82.9% to 97.8%. This trend correlates with the previously discussed structural and physicochemical data: higher synthesis pH for Ag_2_SeO_3_ promotes both increased Ag^+^ ion release and heightened ROS generation, factors that drive the observed cytotoxic response, particularly at elevated concentrations. While Ag^+^ levels up to 0.22 μg/mL are generally tolerated, the estimated release for high-pH samples exceeds this benchmark, likely explaining the pronounced toxicity of ASOpH12.^70^ Notably, the nontoxic concentrations identified here are substantially lower than those required for effective photocatalysis and antimicrobial action. This discrepancy suggests that direct application of these materials in biological environments may be restricted, highlighting the necessity for postprocess separation after photocatalytic treatments.

**Figure 8:**
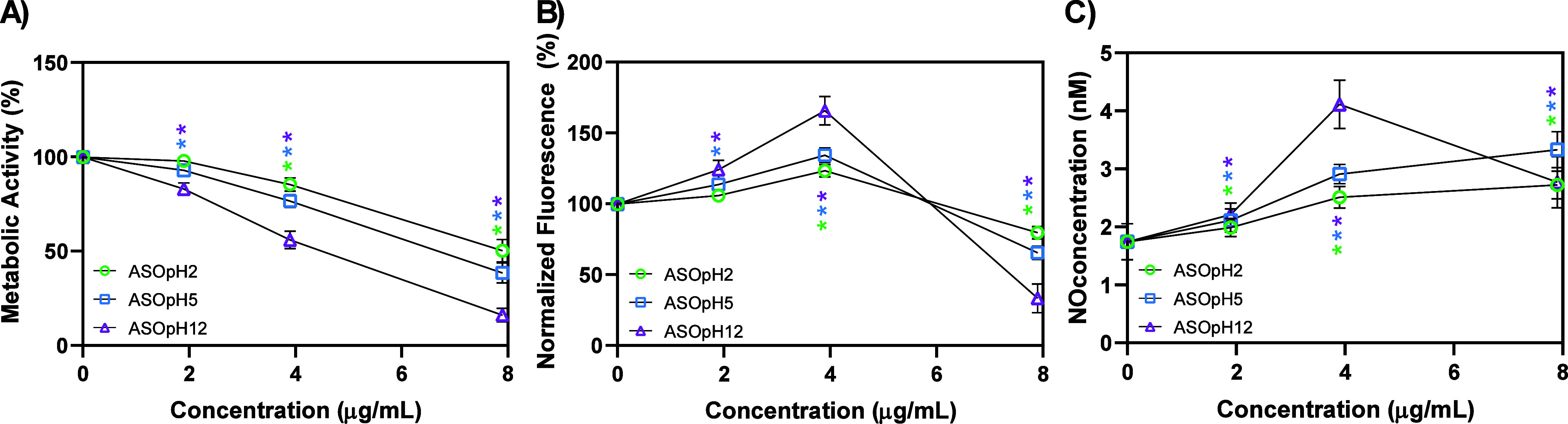
—(A) Metabolic activity of L929 cells assessed by the MTT assay after 24 h of exposure, expressed as percentage of cell viability relative to the control group. (B) Intracellular ROS levels quantified by DCFDA fluorescence. (C) Intracellular RNS production determined by the Griess reaction. Results are presented as mean ± standard deviation (*n* = 9). Statistical significance was determined using one-way ANOVA followed by Tukey’s post hoc test, with *p* ≤ 0.05 (*). All experiments were conducted in triplicate on three independent occasions (*n* = 9).

Intracellular redox responses, quantified via DCFDA fluorescence (ROS) and the Griess reagent (RNS), followed a clear concentration-dependent trend across all Ag_2_SeO_3_ samples (Figure 8b,c). At the minimum dosage (1.9 μg/mL), both ASOpH2 and ASOpH5 maintained ROS/RNS levels comparable to the control group. In contrast, ASOpH12 induced a slight elevation in these parameters, suggesting an early triggering of redox activity that, at this stage, does not breach the 75% viability threshold. The intermediate concentration (3.9 μg/mL) marked a significant shift, with a sharp rise in ROS and RNS production for all materials. This surge was particularly aggressive for ASOpH12, aligning with the metabolic decline observed in the MTT assays. While ASOpH2 and ASOpH5 remained within safe biological limits, the intensified oxidative stress from ASOpH12 highlights how alkaline synthesis conditions enhance Ag^+^ release and subsequent radical generation. Interestingly, at the maximum concentration (7.9 μg/mL), a decline in ROS/RNS signals was recorded. Rather than a true reduction in reactive species, this drop reflects the severe depletion of the viable cell population at this exposure level, leaving fewer functional cells to respond to the probes. Collectively, these findings confirm that Ag_2_SeO_3_-induced cytotoxicity is driven by a synergistic oxidative-nitrosative stress mechanism, directly dictated by ROS production and Ag^+^ leaching.

## 4 Conclusions

This study demonstrates that synthesis pH is a decisive parameter controlling the structural order, defect chemistry, morphology, and multifunctional performance of sonochemically synthesized Ag_2_SeO_3_. Acidic conditions favor well-defined microrod architectures with higher lattice stability and intrinsic structural defects, whereas alkaline synthesis induces pronounced short- and medium-range disorder, preferred crystallographic orientation, oxygen-related defects, and partial metallic Ag segregation. These changes directly modulate charge-carrier dynamics, suppress recombination, and promote efficient ROS generation.

Among the investigated samples, Ag_2_SeO_3_ synthesized at pH 12 exhibited the highest photocatalytic efficiency toward ciprofloxacin degradation, driven primarily by diffusible ^•^O_2_H and ^1^O_2_ species rather than surface-confined ^•^OH radicals. Importantly, photocatalytic residues were rendered nontoxic toward bacteria and *L. sativa*, highlighting effective detoxification despite incomplete mineralization. Antibacterial assays revealed pH-dependent selectivity, with acidic samples showing enhanced activity against Gram-positive bacteria, while alkaline samples favored Gram-negative inhibition due to increased Ag^+^ release and ROS diffusivity.

Biological assays confirmed that cytotoxicity and intracellular oxidative stress scale with defect density and silver ion release, emphasizing the necessity of catalyst recovery after treatment. Notably, the concentrations required to achieve in vitro antimicrobial inactivation were lower than those inducing significant mammalian cell toxicity, indicating a reduced therapeutic window and reinforcing the need for controlled dosage and exposure conditions. Therefore, despite its strong antimicrobial potential, Ag_2_SeO_3_ should be applied under carefully regulated operational parameters, particularly in biomedical or direct-contact applications, to avoid unintended cytotoxic effects. Overall, these findings establish a clear structure–defect–function relationship in Ag_2_SeO_3_ and demonstrate that pH-controlled sonochemical synthesis is a powerful and scalable approach for tailoring silver–selenium semiconductors toward advanced photocatalytic and antimicrobial applications.

## Supplementary Material



## Data Availability

The data supporting this study are available within the manuscript.

## Notes

Se 3p high-resolution spectra, EDS analysis, band structure, and recyclability tests (PDF)
