# Supplementary material for: Defect-Tailored Ag2SeO3: Morphology and Function Tuned by pH-Driven Sonochemical Synthesis
Source: ACS Omega. 2026 Mar 19;11(12):19848–63. doi: 10.1021/acsomega.6c00644 (PMC13044833; doi:10.1021/acsomega.6c00644)
Supplement: Supplementary file 1 [file ao6c00644_si_001.pdf]

Supplementary Information

Defect-Tailored Ag_2SeO_3: Morphology and Function Tuned by pH-Driven Sonochemical Synthesis

Henrique Moreno[a], Giovanna A. Grasser[a], Marcio D. Teodoro[b], Marcelo Assis[c], Elson Longo[a]

[a] CDMF-LIEC,Federal University of São Carlos (UFSCar), São Carlos, 13565-905, Brazil.

[b] Department of Physics, Federal University of São Carlos (UFSCar), São Carlos, 13565-905, Brazil.

[c] Department of Biosciences, Federal University of São Paulo (UNIFESP), Santos, SP 11015-020, Brazil

*Corresponding author: hpiccolimoreno@gmail.com

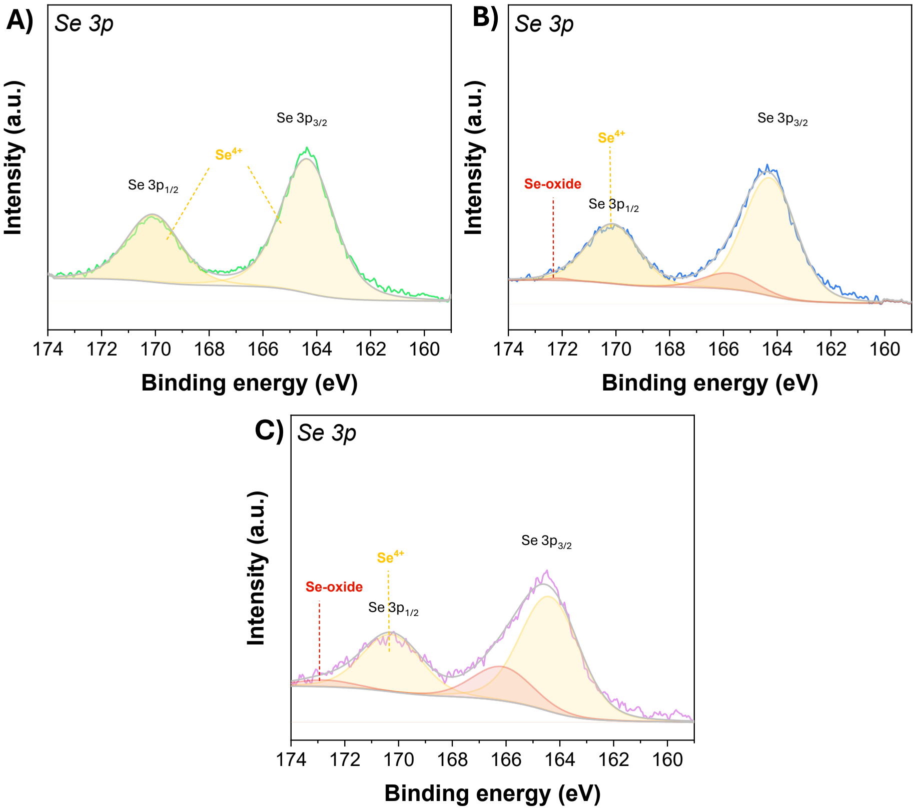

Figure S1 – Se 3p high-resolution XPS spectra obtained for the samples (A) ASOpH2, (B) ASOpH5 and (C) ASOpH12.

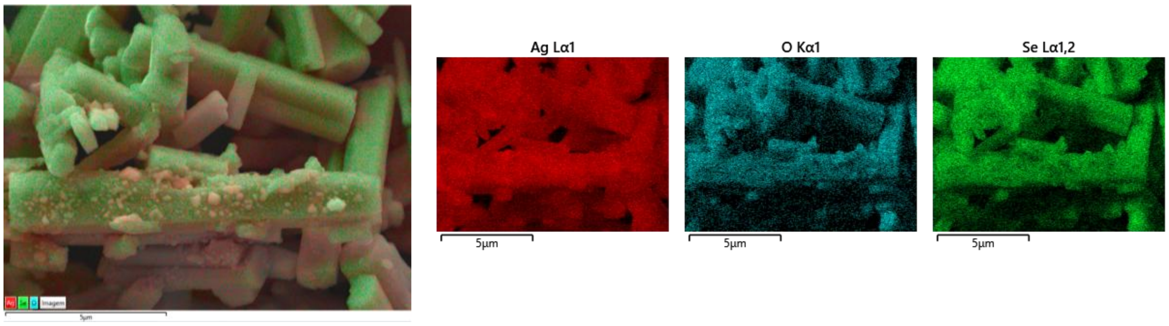

(a) ASOpH2

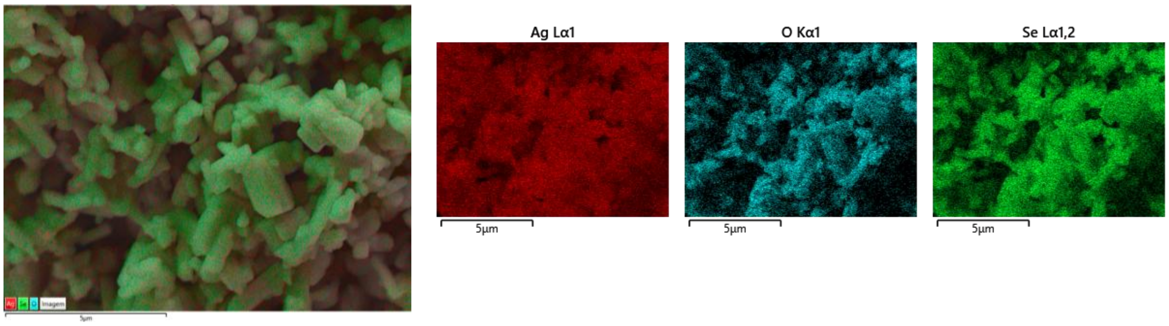

(b) ASOpH5

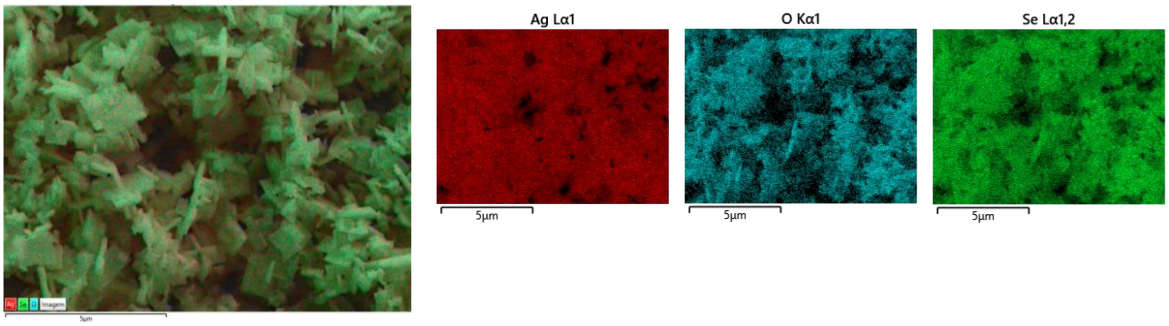

(c) ASOpH12

Figure S2 – Elemental maps obtain by SEM for the samples (a) ASOpH2, (b) ASOpH5 and (c) ASOpH12.

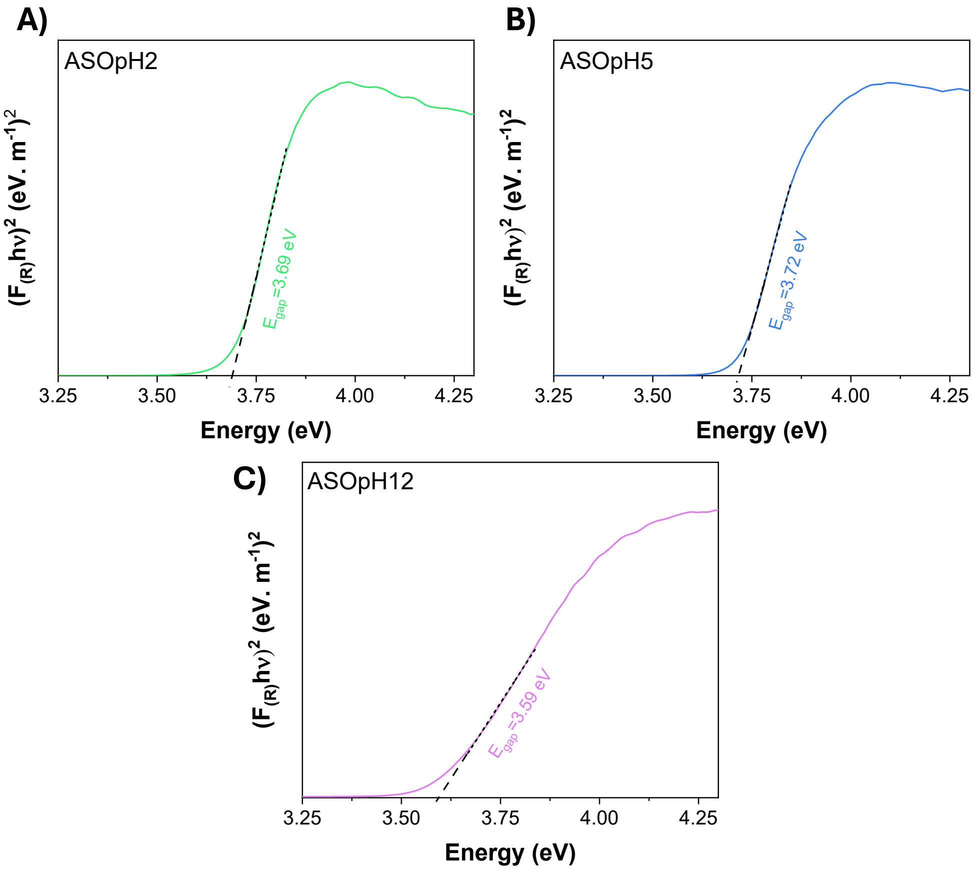

Figure S3 – Kubelka-Munk Tauc plots obtained for samples (a) ASOpH2, (b) ASOpH5, and (c) ASOpH12 with their respective estimated E_{gap} values.

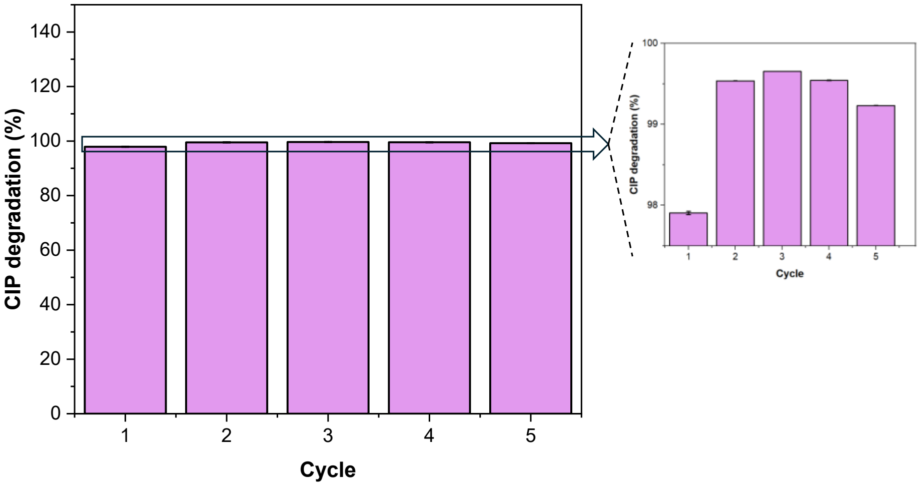

Figure S4 – Recyclability tests performed for sample ASOpH12 over 5 subsequent degradation cycles. *All measurements were performed in triplicates.

Table S1 – Synthesis' parameters used for each of the samples considering pH control. *pH was measured using a pHmeter at room temperature.

Sample	[$AgNO_3$] (M)	[SeO_2] (M)	pH_0	pH_f	V_0 (mL)	V_{NaOH} (mL)	V_f (mL)
ASOpH2	0.8	0.2	2	2	75	-	75
ASOpH5	0.8	0.2	2	5	75	4	79
ASOpH12	0.8	0.2	2	12	75	10	85
